# Article biodiversity inside bottles: animals, fungi, and plants in traditional alcoholic drinks

**DOI:** 10.3389/fnut.2024.1368110

**Published:** 2024-04-19

**Authors:** María Cruz Juárez-Aragón, Yolanda del Rocio Moreno-Ramírez, Reyna Ivonne Torres-Acosta, Jorge Ariel Torres-Castillo

**Affiliations:** ^1^Unidad Académica de Trabajo Social y Ciencias para el Desarrollo Humano, Universidad Autónoma de Tamaulipas, Ciudad Victoria, Tamaulipas, Mexico; ^2^Facultad de Ingeniería y Ciencias, Universidad Autónoma de Tamaulipas, Ciudad Victoria, Tamaulipas, Mexico; ^3^Unidad Académica Multidisciplinaria Mante Centro, Universidad Autónoma de Tamaulipas, Ciudad Mante, Tamaulipas, Mexico; ^4^Instituto de Ecología Aplicada, Universidad Autónoma de Tamaulipas, Ciudad Victoria, Tamaulipas, Mexico

**Keywords:** drinks, plant extract, bioactive compounds, antioxidants, native foods

## Abstract

The use of animals, fungi, and plants as a source of bioactive compounds has been widely practiced in diverse cultures throughout the world, particularly in alcoholic drinks. The nature of the biological material, method of preparation and alcohol concentration play a predominant role in the extraction of bioactive compounds and the achievement of desired results. However, certain aspects must be considered to guarantee the innocuity of these drinks and reduce the risk of intoxication, infections and allergic reactions, aspects which are sometimes overlooked. In addition, the implications of using threatened or protected species must be considered to reduce the negative impact on their populations. The authors recommend the establishment of production systems which guarantee products with adequate quality controls and ensure the benefits to the consumer.

## Graphical abstract

Biodiversity in bottles.

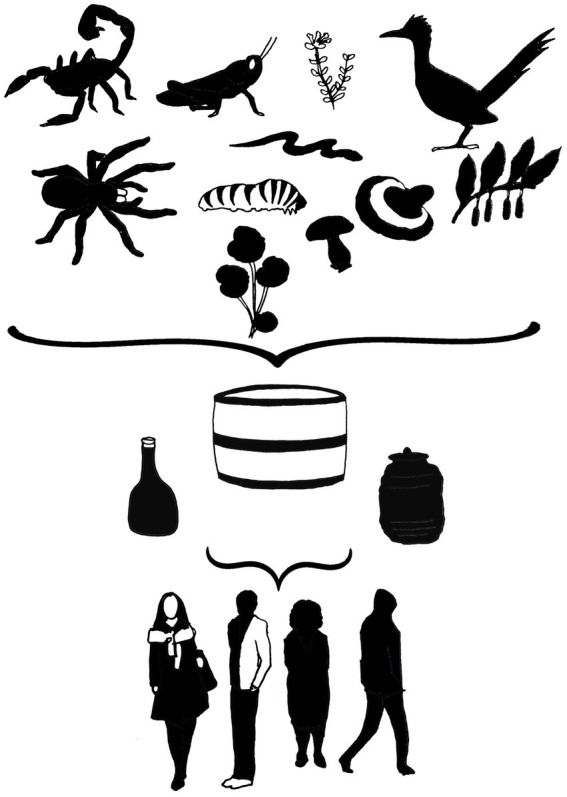

## 1 Introduction

Thousands of bottles containing alcoholic drinks have withstood the passage of time and diverse events (shipwrecks, migrations, changes in political and cultural power, among others) preserving within them their chemical composition and the resources used in their production, which together serve as unique chemical fingerprints revealing their past and potential future. The use of biotic resources to meet nutritional and health needs throughout human history has given rise to specific methods of preparation and consumption linked to social, cultural, economic, and political development and in distinct geographical spaces and times (1). Alcoholic preparations to which bioactive properties are attributed have been part of traditions and cultural identity for centuries, although their functional principles (antioxidant, relaxant, anti-inflammatory, analgesic, anticancer, neuroactive) and the characterization of the organisms used in their preparation, for the most part, remain to be explored. In this sense, traditional alcoholic drinks which are fortified with plants, animals, and fungi—usually used as part of the culinary, medical, and cultural repertoire—provide benefits to the consumer which, in many cases, has limited the use of these organisms to commercial attraction, that is, a marketing strategy (2). Although for the most of these alcoholic drinks, no clinical evidence has demonstrated such bioactive benefits, their consumption has been kept through the time. Nevertheless, the incorporation of these organisms can have greater significance due to their health benefits, contribution to food security, benefits to rural tourism, social and religious value as well as their role in the preservation and transmission of cultural heritage (3). All the above and consumer literacy of this knowledge can expand the use of these traditional drinks beyond solely recreational purposes. However, it is important to ensure product quality and the benefits to the consumer and consider regulations regarding their consumption and the sustainable use of species which become part of these alcoholic drinks rich in bioculture and potential.

## 2 Diversity in preparations

The solvents most often used in the preparation of alcoholic drinks are ethanol solutions in varying concentrations. In some such drinks, the percentage of alcohol varies between 30 and 40% according to the general content for most spirits, although concentrations of some may be as high as 75%, while some are only 14% (4–6). Preparation time is variable, and the surrounding operations are diverse; Armor Tail Scorpion Vodka from the Thai brand Unique®, for example, is infused with a scorpion (3 to 4 cm in length) for several months (Thailand Unique Trademark). The maguey worm (*Comadia redtenbacheri* Hammerschmidt) is incorporated into bottles of mezcal (distilled form Mexican agave), a simple process which has been diversified. One way is to submerge *C. redtenbacheri* in water at 80° C before adding them to white vinegar and 40% ethyl alcohol at a ratio of 1:1 for 24 h. The worms are then put in 40% alcohol for 7d before being incorporated into the bottles of mezcal (7), where they can remain for months and even years. In Korea, fermentation is a favored technique in alcohol production for the addition of plant species. Some 50% of drinks are prepared in specific seasons depending on the availability of ingredients (flowers, fruits, roots, and leaves) and for several purposes associated with celebrations and religious practices (8). The above illustrates the wide variability of base drinks and the procedures for preparing traditional liquors in different parts of the world (Table 1).

**Table 1:** Examples of alcoholic drinks with recipes and associated bioactive properties.

Drink (Country)	Recipe	Bioactive properties	Clinical trials	Reference
Snake wine (India, Vietnam)	First, an adult snake of medicinal, venomous, or not venomous species is not feed for 2 to 3 days. Afte this, the individual is immersed it alive into recipient with rice wine in a ratio 1:10 (w/v). Second, the snake is basically left release venom and after this, the snake body is retired. Some species like spices, herbs, roots, ferns, seeds, honey, scorpions, or insects are included to increase flavors, colors, and healing properties. This preparation is reposed for 1 year before use. An alternative includes the use of 50–200 g of snake flesh, in a 1: 5 (w/v) ratio. The preparation starts with cleaning of flesh, then it is immersed in rice wine or 60% liquor. Rest is usually don by 3 months before use. Some species are recommended: copper head snake, coral snakes, cobras snakes, and rattle snake.	Anti-inflammatory, analgesic, antibacterial, sight improvement, sexual enhancement, fever relieve.	No	Lachenmeier et al. (5) and Parashar and Panwar (9)
Latifa drink (Azerbaijan)	First, plant syrup preparation (based on *Rumex sculatus* spp. *hastifolicus* (M. Bieb), Peppermint (*Mentha piperita* L.), plantain (*Plantago* sp. L.) *Hipericum perforatum* L): Dry plant material (1:15, w/v) is extracted in water at 85°C for 1 h, then mixture is cooled to 50–55°C and treated for 30 min with a pectolytic enzyme and then filter. Extract must content 2–3% of dry matter to be mixed with sugar and citric acid in ratio 44.39:55.44:0.17 and heated at 50°C with continuous stirring until all is dissolved. Then mixture is boiled to reduce volume to 70–71% with stirring during 2 h and cooled at 20°C. After this, syrup is filtered to be ready to use. Second, the aromatic fraction: An extract of the pear juice of Latifa variety (57.4% of dry matter content) and infusions of feijoa (*Acca sellowiana* (O. Berg.) Burret), lemon and nuts of the milk-wax stage in a ratio 33:33:22:12 Infusions are prepared by crushing nuts in 50% ethanol using a ratio 1:10 (w/v) for 10 days, stirring each day. After, this infusion is recovered by filtration and store between 10 to 15°C until 6 months, to preserve its aromas. In the case of feijoa, fruits are filled with 68.2% ethanol in a ratio 1:1 and lemons are filled with 76% ethanol in a ratio 1:5 (w/v). Preparation is done by mixing 85.5% of prepared water (with a hardness of no more than 1,426 mg. eq / L), 10% extractive part and 4.5% of aromatic part.	Antioxidant	No	Gharib et al. (10)
Flavored alcoholic beverages (Romania)	Preparation is done by macerated plant material in vodka (ethanol 37.5%) in a ratio 1:25 (w/v) for 72 h at room temperature. Addition of sugar syrup in a ratio 1:2 (w/v) was used to improve taste and color of alcoholic drinks. Suggested plant material included: elderberry flower, mint, thyme, cinnamon, rosemary, and lemon balm.	Antioxidant	No	Moigradean et al. (11)
Yakju (Korea)	Firstly, 40 g of non-glutinous rice are incorporated to 50 mL of boiling water and then, they are heated for 10 min. After, mixture must be cool, followed by addition of 10 g of koji, 5 g of cooked rice flour and one milliliter of *S. cerevisiae* (grown in YEPD medium at 30°C for 2 d were added). This mixture is left resting for two days at 25°C. After this, 50 g of glutinous rice and 50 g of non-glutinous rice are heated at 100°C for 1 h and then, cooled to room temperature. To this rice, 100 mL of water, the initial first mixture and 1% of Ganoderma lucidum powder are incorporated and fermented during 10 days at 25°C. Finally, all this mixture is filtered using a cotton cloth or centrifuged to obtain the yakju.	Antioxidant Gastroprotective effect	No	Kim et al. (12)
Ganoderma infusion (Serbia)	Preparation initiates with maceration of small pieces (around 1 cm) of fruiting body of *Ganoderma* species (*G. lucidum*, mainly) in aqueous solution of 40–70% preferent to 45–60%. With a recommended extraction conditions of 25 g/L during 21 days at room temperature, followed by filtration.	Treatment of multiple diseases	Yes	Veljović et al. (13)
Whole-scorpion wine (China)	Preparation includes two liquid components: First 24 kilograms of alive scorpions are submerged in 120 liters of 39° grain Chinese liquor during a week, after this, scorpions are retired, and liquid is recovered. Second, in 60 liters of 50° grain Chinese liquor, 240 g of Chinese cassia trees and 80 g of dried orange peels are immersed for 24 h. After this, liquid is recovered. Whole-scorpion wine is prepared by mixing previous preparations in a ratio 1:1.	Treatment of multiple diseases	No	Sun et al. (14)

## 3 Drinks with arthropods and reptiles

The use of arachnids (mainly tarantulas and scorpions) in alcoholic drinks is to increase the visual impact on the consumer, and the practice has been broadly developed in a variety of traditional drinks, such as rice wine in the case of the Thai brand, Unique®, for scorpion wine in Vietnam, which is a product widely recognized in Asia (15, 16). The elaboration of mezcal has been part of Mexican culture since the 17th century and one of the most popular mezcals is tequila, designated with this name based on the use of *Agave tequilana* Weber. However, it was not until the 1940s and 50s that the mezcal worm was incorporated and became identified as the tequila worm, increasing public interest in mezcals, especially in Asia, Europe, and the United States (17). Many myths have arisen around the addition of *C. redtenbacheri* to mezcals, such as its aphrodisiac properties; however, since there is no evidence to support the claim, it is considered more marketing than functionality. Nevertheless, the addition has driven the incorporation of other arthropods, such as scorpions. Scorpions are used based on their availability and distribution in the region and their incidence in the area where the mezcal is prepared. For example, mezcal produced in the state of Durango, Mexico includes scorpions from the genus *Centruroides* spp., while some Oaxacan mezcals include species from the genera *Centruroides* and *Vaejovis*, giving resinous notes to the drink (18). While the above speaks to recreational use, the incorporation of arthropods to mezcal for medicinal purposes is practiced by ethnic groups in Central Mexico, such as in the case of centipedes from the genus *Scolopendra*, which are used as a traditional remedy against poisonous animals (19). The addition of insects to increase antioxidant levels in alcoholic drinks and provide them with sensory attributes in a controlled way is an innovative contribution to generate products with attainable quality criteria, as indicated with the incorporation of *Pterophylla beltrani* Bolívar and Bolívar., *Schistocerca piceifrons* Walker and *Tenebrio molitor* L. (6, 20). More extravagant is the introduction of snakes, geckos, birds, and other animals into some alcoholic mixtures, ostensibly for visual impact and health benefits. Snake wine, from Vietnam, is perhaps the best known and most striking exotic drink, with the use of venomous species including cobras. Meanwhile, in Mexico, the incorporation of rattlesnake into sotol (a distilled beverage from the fermentation of the stem of *Dasylirion* spp.) has been reported (21, 22). A wide range of practices and production processes are carried out in the different regions of origin of these resources (plants and animals) and production. Within each region, the incorporation of different animal groups shows the convergence between culture and biodiversity, that is, the bioculture surrounding the traditional alcoholic drinks recognized as icons of the state and country’s tradition.

## 4 Drinks with fungi

Fungi are widely recognized around the world as important sources of antioxidant and neuroactive compounds. They are associated with benefits such as analgesic and neuroprotective properties, in the treatment of nervous, circulatory, and digestive diseases, cancer and diabetes (23); their use to fortify alcoholic drinks with medicinal attributes is common and highly represented in Chinese medicine (13), and they have earned certain popularity in other parts of the world. The most popular fungi used in the elaboration of this kind of drink is the *Agaricomycete* group, which has shown numerous beneficial effects associated with its bioactive compounds (24–26). The fungus *Lignosus rhinocerus* (Cooke) Ryvarden has been indicated to treat wounds, asthma, fever, cancers and food poisoning; *L. rhinoceros* powder is also mixed with Chinese rice wine and, surprisingly, can be applied topically to treat lumps, sores and boils (27). On the other hand, the fungus *Hericium erinaceus* (Bull.: Fr.), known as lion’s mane, is associated with a decrease in neurodegenerative diseases. Commonly mixed with a made wine, it is highly recommended for its regenerative effects on the nervous system; in fact, an alcoholic preparation of *H. erinaceus* is sold by Fungi Perfecti Co. (28). Perhaps the most popular fungus as a source of antioxidants and used medicinally by infusing in alcohol is *Ganoderma lucidum* (Curtis) P. Kart., which owes its antioxidant and antiaging properties to its metabolites, mainly terpenoid and phenolic compounds, and much information exists related to the technical aspects of its production, handling, and preparation, as well as its bioactive effects, in fact, it is the only fungus which an alcoholic preparation has been submitted to clinical trials (14, 29). The fungus *Cordyceps sinensis* (Berk.) Sacc., belonging to the Ascomycetes, has been widely used for its medicinal and invigorating properties, but has its origins in traditional Chinese medicine. Its consumption as a supplement in alcoholic drinks has mainly been as a tonic or an ingredient mixed with local alcoholic drinks which are left to rest for around an hour before consuming (30). The use of fungi in the preparation of alcoholic drinks is versatile and can be used in various contexts (medicinal, recreational, ceremonial), which determines the production method and technical degree involved, from traditional to more elaborate and well-researched methods to achieve mass production. Cordyceps strains have been evaluated in human studies as capsules treatment and results have shown health and fitness improvement; however, no alcoholic drinks have been reported with clinical trials (31–33).

## 5 Drinks with plants

Alcoholic drinks with medicinal purposes which incorporate plants are very diverse and, as in the previous cases, their fortification will depend on the cultural practices and prevailing vegetation in the ecosystem, and in some cases, seasonality, and festivities (8). Wu Jia Pi wine is perhaps one of the most famous medicinal liquors and has its origin in traditional Chinese medicine. The drink is attributed with properties which aid in blood circulation, invigorate the body, and reduce fatigue. Its preparation includes nine species of medicinal plants containing alkaloids, antioxidants, and essential oils, notably *Gardenia jasminoides* J. Ellis*, Polygonatum odoratum* (Mill.) Druce, *Cortex* acanthopanacis (*Acanthopanax gracilistylus* W. W. Smith), Vladimirae Radix root (*Vladimiria souliei* (Franch.) Ling), Fructus lycii (*Lycium chinense* Mill.), *Cinnamomum cassia* (L.) J. Presl, *Angelica sinensis* (Oliv.) Diels., Fructus amomi (*Amomum vilosum* Lour.), and the spice *Syzygium aromaticum* ((L.) Merr. and L. M. Perry). The liquor has even been produced industrially, sustained by the beneficial effects that have been promoted traditionally for centuries (34). Another example is the consumption of damiana liqueur (*Turnera diffusa* Willd. ex Schult.), which is related to toning and relaxing, as well as hypoglycemic and supposedly aphrodisiac effects brought on by compounds such as flavonoids, which are highly soluble in the hydroalcoholic mixture. The plant is widely used across much of Latin America; in Mexico, it is mixed with tequila or mezcal to prepare relaxing and sexually stimulating drinks (35). Traditionally, the consumption of alcoholic drinks as part of the gastronomy and customs in southern Europe and the Mediterranean involves a variety of aromatic and medicinal species, giving rise to diverse flavors, aromas and biofunctional compounds. The diversity of plants recorded as raw materials for the fortification of functional alcoholic drinks includes mainly members of the Lamiaceae, Asteraceae, Rosaceae, Rutaceae, and Apiaceae families. Their preparation involves procedures such as maceration and codistillation, and they can be consumed in a variety of ways, as recreational or accompanying drinks or as traditional medicines (36). In particular, the flavonoids as a conspicuous phytochemical group, are present in many plants-derived alcoholic drinks, and although no strong clinical evidence about benefic effects of alcoholic drinks themselves, flavonoids have been associated with beneficial health effects like anticancer, promotion of cognitive functions, prevention of hypertension and antidiabetic activities by clinical trials (37–40), which suggests the need for research on beneficial effects promoted by plant-derived alcoholic drink consumption. Clearly, flora as a natural resource base to produce fortified drinks is associated with traditional practices that are closely linked to the ecosystem and resource availability, and which have been used for centuries and are part of our cultural repertoire. However, it is important to consider the technical issues regarding the preparations between regions or for formulas developed for home use or on an industrial level, to preserve the properties which benefit the consumer.

## 6 Points to consider

The consumption of the traditional alcoholic drinks is a common practice, which, depending on the case, can be traced back for centuries, an indication that these products are deeply rooted in the culture of the people. However, the inherent risks associated with their preparation and consumption cannot be ignored (36). Products which originate from official establishments with a tradition of handling such drinks may be the best option, although the effects claimed in most cases remain to be confirmed. Risks have reported regarding the authenticity, origin and harmlessness of the specimens used to prepare some of these drinks, as in the case of snake wine, which incorporates snakes of uncertain origin (21); or the scorpions added to drinks in Mexico, which are collected in the wild with no guarantee their safety or composition (18). It is also worth mentioning that plants and fungi must be correctly identified to avoid possible intoxications, as well as to ensure the desired effect. Alcohol content is another important point which, depending on the material to be incorporated into the base preparation, should be high enough to promote extraction, reduce microbial growth and adequate to avoid intoxication if consumed in excess (5). As many of these products are traditional in nature, the majority lack registration, labelling or content specifications of bioactive substances or raw materials, suggesting risks for consumers and an urgent issue to consider by health authorities. The consumption of traditional alcoholic drinks of this kind would appear common and, to a point, harmless and recreational. Nevertheless, serious health situations can occur, such as intoxication, allergy and even death, as reported in the consumption of snake wine, which caused severe coagulopathy (41), or anaphylaxis on consuming the fungus *H. erinaceus* (42) or the high level of fluoride that accumulates in black tea and could lead to excess fluoride in the human body if consumed in excess (43). Above all, it is vitally important that the production of such drinks considers the vulnerability of the natural populations of the species collected, to avoid threatening the biodiversity and promote their permanence over time.

## 7 Conclusion

Traditional alcoholic drinks have maintained the same time-honored ingredients and characteristic preparation methods and techniques which give them identity and reflect their bioculture with specific properties that distinguish them from others. They are an important part of cultural heritage, upholding an historic-cultural legacy linked to biotic resources from their region of origin/distribution. The incorporation of various biotic components from the ecosystem promotes both their existence and the value of their chemical composition, which for decades, even centuries, has facilitated the inclusion of bioactive compounds in the human diet. However, gaps in knowledge, a lack of health regulations and the unsustainable use of some of the species used, explain the urgency to introduce sustainable practices for the use and preservation of all their elements (plants, animals, and fungi), including the bioculture relevance of those alcoholic drinks prepared using traditional knowledge. More research is needed to ensure the quality, quantity, bioavailability, and clinical support for benefits of their consumption.

## Author contributions

MJ-A: Conceptualization, Investigation, Writing – original draft, Writing – review & editing. YM-R: Investigation, Validation, Writing – original draft, Writing – review & editing. RT-A: Writing – original draft, Writing – review & editing. JT-C: Conceptualization, Investigation, Supervision, Validation, Writing – original draft, Writing – review & editing.
